# Preclinical Characterization of a Stabilized Gastrin-Releasing Peptide Receptor Antagonist for Targeted Cancer Theranostics

**DOI:** 10.3390/biom13071134

**Published:** 2023-07-14

**Authors:** Ayman Abouzayed, Panagiotis Kanellopoulos, Alisa Gorislav, Vladimir Tolmachev, Theodosia Maina, Berthold A. Nock, Anna Orlova

**Affiliations:** 1 Department of Medicinal Chemistry, Uppsala University, 751 83 Uppsala, Sweden; ayman.abouzayed@ilk.uu.se (A.A.); panagiotis.kanellopoulos@ilk.uu.se (P.K.); alisa.gorislav@ilk.uu.se (A.G.); 2 Molecular Radiopharmacy, INRaSTES, NCSR “Demokritos”, 15310 Athens, Greece; maina_thea@hotmail.com (T.M.); nock_berthold.a@hotmail.com (B.A.N.); 3 Department of Immunology, Genetics and Pathology, Uppsala University, 751 83 Uppsala, Sweden; vladimir.tolmachev@igp.uu.se; 4 Science for Life Laboratory, Uppsala University, 752 37 Uppsala, Sweden

**Keywords:** prostate cancer, GRPR antagonist, theranostics, PC-3 cells, neprilysin

## Abstract

Radiolabeled gastrin-releasing peptide receptor (GRPR) antagonists have shown great promise for the theranostics of prostate cancer; however, their suboptimal metabolic stability leaves room for improvements. It was recently shown that the replacement of Gly^11^ with Sar^11^ in the peptidic [D-Phe^6^,Leu^13^-NHEt,*des*-Met^14^]BBN(6–14) chain stabilized the [^99m^Tc]Tc-DB15 radiotracer against neprilysin (NEP). We herein present DOTAGA-PEG_2_-(Sar^11^)RM26 (AU-RM26-M1), after Gly^11^ to Sar^11^-replacement. The impact of this replacement on the metabolic stability and overall biological performance of [^111^In]In-AU-RM26-M1 was studied using a head-to-head comparison with the unmodified reference [^111^In]In-DOTAGA-PEG_2_-RM26. In vitro, the cell uptake of [^111^In]In-AU-RM26-M1 could be significantly reduced in the presence of a high-excess GRPR-blocker that demonstrated its specificity. The cell uptake of both radiolabeled GRPR antagonists increased with time and was superior for [^111^In]In-AU-RM26-M1. The dissociation constant reflected strong affinities for GRPR (500 pM for [^111^In]In-AU-RM26-M1). [^111^In]In-AU-RM26-M1 showed significantly higher stability in peripheral mice blood at 5 min pi (88 ± 8% intact) than unmodified [^111^In]In-DOTAGA-PEG_2_-RM26 (69 ± 2% intact; *p* < 0.0001). The administration of a NEP inhibitor had no significant impact on the Sar^11^-compound (91 ± 2% intact; *p* > 0.05). In vivo, [^111^In]In-AU-RM26-M1 showed high and GRPR-mediated uptake in the PC-3 tumors (7.0 ± 0.7%IA/g vs. 0.9 ± 0.6%IA/g in blocked mice) and pancreas (2.2 ± 0.6%IA/g vs. 0.3 ± 0.2%IA/g in blocked mice) at 1 h pi, with rapid clearance from healthy tissues. The tumor uptake of [^111^In]In-AU-RM26-M1 was higher than for [^111^In]In-DOTAGA-PEG_2_-RM26 (at 4 h pi, 5.7 ± 1.8%IA/g vs. 3 ± 1%IA/g), concordant with its higher stability. The implanted PC-3 tumors were visualized with high contrast in mice using [^111^In]In-AU-RM26-M1 SPECT/CT. The Gly^11^ to Sar^11^-substitution stabilized [^111^In]In-DOTAGA-PEG_2_-(Sar^11^)RM26 against NEP without negatively affecting other important biological features. These results support the further evaluation of AU-RM26-M1 for prostate cancer theranostics after labeling with clinically relevant radionuclides.

## 1. Introduction

Prostate cancer is one of the most commonly diagnosed cancers in men and the cause of many cancer-related deaths [1]. A lot of effort has been focused on targeting prostate cancer cell markers, such as the prostate-specific membrane antigen (PSMA) and gastrin-releasing peptide receptor (GRPR), to improve diagnosis and therapy. Owing to its overexpression in the vast majority of prostate cancer lesions [2], PSMA has been extensively studied and a number of FDA-approved PSMA-radioligands are currently commercially available for the management of PSMA-avid prostate cancer [3,4]. However, a number of limitations have become evident during the clinical application of PSMA-targeting radioligands in recent years. Firstly, resistance to PSMA-targeted radionuclide therapy has been reported [5]. Secondly, PSMA expression is frequently suboptimal in low Gleason score tumors [6] and can also be suppressed in some advanced and aggressive forms of prostate cancer, such as neuroendocrine prostate cancer [7]. Therefore, the investigation of prostate cancer cell markers beyond PSMA is still valid, including GRPR as an example.

GRPR is a G-protein-coupled receptor involved in the regulation of several physiological body functions, such as gastric acid secretion and gastric motility [8,9,10]. It is endogenously expressed in several human organs and tissues, including the pancreas, stomach, and intestinal wall [11]. The GRPR is also highly expressed in several malignancies, including prostate, breast, and colon cancer. GRPR overexpression has been documented in 63–100% of primary prostate cancer samples and in the majority of lymph node and bone metastases, while most benign and hyperplastic prostate cells are GRPR negative [12,13,14,15]. Further, GRPR expression in prostate cancer is higher in the earlier stages of the disease and is androgen-dependent [16,17,18]. Hence, GRPR-targeting may play an important complementary role to PSMA-targeting, for example, in the management of oligometastatic prostate cancer.

Several GRPR radioligands have been developed and evaluated in preclinical and early-phase clinical studies in prostate cancer patients, demonstrating great potential and safety for theranostic application. The issue of safety has been vigorously addressed by the advent of GRPR antagonists that, unlike agonists, do not elicit acute adverse effects after binding to the endogenous receptors. Accordingly, higher doses of GRPR antagonists can be safely injected into patients without inducing adverse effects or cancer cell proliferation [19,20] as well as the undesirable downregulation of GRPR [21]. Moreover, radiolabeled GRPR antagonists have frequently displayed superior pharmacokinetics compared to agonists, combining a higher tumor uptake with a faster body clearance, resulting in higher tumor-to-background ratios [22,23].

The degradation of peptide-based radioligands with proteases after injection negatively affects tumor uptake and overall biodistribution profiles [24]. The action of proteases, such as neprilysin (NEP), on GRPR radioantagonists was shown to impair their bioavailability compromising the tumor uptake [25,26]. Other studies, which investigated the effect of NEP inhibition on the radioligand stability, demonstrated an increased tumor uptake and an improved therapeutic outcome in animal models [25,27]. A lot of time and effort has been invested to improve the metabolic stability of radiolabeled GRPR antagonists. Thus, a good number of structural interventions was undertaken for enhancing resistance to the proteolytic action of implicated proteases, especially NEP [28].

Our group has been extensively working on radioligands based on the GRPR antagonist JMV594 motif (or RM26, [D-Phe^6^,Sta^13^,Leu^14^-NH_2_]BBN(6–14)) [29]. In one of our earlier studies, we conjugated RM26 to DOTAGA chelator (1,4,7,10-tetraazacyclodocecane,1-(glutaric acid)-4,7,10-triacetic acid) via a PEG_2_ linker (DOTAGA-PEG_2_-RM26) and radiolabeled it with In-111. During preclinical evaluation, [^111^In]In-DOTAGA-PEG_2_-RM26 demonstrated high GRPR affinity and a favorable biodistribution profile [30]. In a following study, the uptake of [^177^Lu]Lu-DOTAGA-PEG_2_-RM26 in GRPR-expressing tissues significantly increased during NEP inhibition [31], a fact that was previously shown to improve the imaging quality and therapeutic outcome [25,27].

**Figure 1:**
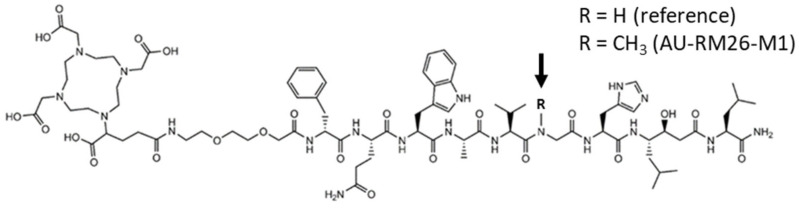
Chemical structures of DOTAGA-PEG2-RM26 (R = H) and AU-RM26-M1 (R = CH_3_).

In the present study, we have modified DOTAGA-PEG_2_-RM26 with a Gly^11^ to Sar^11^ substitution (Sar: sarcosine or N-methylglycine; Figure 1) to improve its metabolic stability by obtaining AU-RM26-M1. Such modification in [^99m^Tc]Tc-DB15 (DB15: N_4_-AMA-DGA-_D_Phe^6^,Sar^11^,LeuNHEt^13^]BBN(6–13); N_4_: 6-carboxy-1,4,8,11-tetraazaundecane, AMA: *p*-aminomethylaniline, DGA: diglycolic acid) led to full radioligand resistance to NEP in vivo linked with a high tumor uptake in mice models and in breast cancer patients [32,33]. The impact of this modification on metabolic stability and several other biological features was assessed in a head-to-head comparison of [^111^In]In-AU-RM26-M1 with the unmodified [^111^In]In-DOTAGA-PEG_2_-RM26 reference.

## 2. Materials and Methods

PC-3 cells were purchased from ATCC (Manassas, VA, USA). Roswell Park Memorial Institute (RPMI) 1640 medium supplemented with L-Glutamine was purchased from Biowest (Nuaillé, France), fetal bovine serum was purchased from Sigma-Aldrich (St. Louis, MO, USA), and penicillin–streptomycin (10,000 U/mL penicillin and 10,000 μg/mL streptomycin) and trypsin–EDTA were purchased from Biochrom AG (Berlin, Germany). For stability studies, Entresto^®^ pills (200 mg corresponding to 24 mg/26 mg sacubitril/valsartan per pill) containing the prodrug sacubitril (in vivo releasing the potent NEP inhibitor sacubitrilat) [34] were purchased from a local pharmacy. Pills were ground to a fine powder in a mortar, divided, and suspended in tab water to individual 12 mg/200 mL doses of pill per animal administered per os (Entresto^®^ group) [35].

Activity measurements were performed on a 2480 Wizard^2®^ automatic gamma counter from PerkinElmer (Waltham, MA, USA). Instant thin-layer chromatography (ITLC) strips (Agilent Technologies, Santa Clara, CA, USA) were used to estimate the radiochemical yield using a Cyclone^®^ Plus Storage Phosphor System from PerkinElmer (Waltham, MA, USA). Real-time affinity measurements were performed using LigandTracer^®^ from Ridgeview Instruments AB (Uppsala, Sweden). Data analysis was performed by applying the unpaired two-tailed *t*-test of GraphPad Prism 8 (GraphPad, San Diego, CA, USA).

The in vivo targeting specificity, biodistribution over time, and SPECT/CT studies performed were approved by the Ethics Committee for Animal Research in Uppsala (Sweden) and followed the national legislation on the protection of laboratory animals (Ethics permit: 5.8.18-00473/2021, 2021-02-26). The in vivo stability study was conducted in a licensed facility (EL 25 BIO exp021) and the protocol was approved by the Department of Agriculture and Veterinary Service of the Prefecture of Athens (#1609, 24-04-2019).

### 2.1. Cell Culture

PC-3 cells were cultured in RPMI 1640 medium supplemented with 20% fetal bovine serum, 1% penicillin–streptomycin and 1% L-Glutamine. The cells were incubated at 37 °C and in 5% CO_2_ in a humidified atmosphere.

### 2.2. Radiolabeling

DOTAGA-PEG_2_-RM26 and AU-RM26-M1 (both custom-produced by Pepmic Co., Ltd., Suzhou, China) were radiolabeled with indium-111 by adding the relevant peptide (3 nmol in 3 µL) to 40 µL ammonium acetate buffer (0.2 M, pH 5.5), followed by addition of 40 MBq [^111^In]InCl_3_ in 54 µL of stock solution (Curium Pharma, Stockholm, Sweden). The mixture (total volume 97 µL) was then incubated for 30 min at 85 °C. The radiochemical yields were determined using ITLC with citric acid (0.2 M) as mobile phase (Rf = 0 for the radiolabeled peptide and Rf = 1 for free In-111). In addition, analysis of the reaction mixture was performed with reversed-phase, high-performance liquid chromatography (RP-HPLC). The RP-HPLC system used was from Hitachi High-Tech (Tokyo, Japan) using a Luna C18 column (5 μm, 100 Å, 150 × 4.6 mm, Phenomenex, Værløse, Denmark) with a gradient from 5 to 70% acetonitrile (MeCN) (0.1% *v*/*v* trifluoroacetic acid, TFA) in water over 15 min (system 1).

### 2.3. Radiometal Complex Stability

The radiochemical stability to transchelation was assessed by incubation of the radiolabeled peptide either with 1000× molar excess of EDTA or by incubation in human serum for 1 h at 37 °C. Incubation of radiolabeled peptide in PBS was used as control. The leakage of radiometal from the radiometal-chelate was determined with ITLC.

### 2.4. In Vivo Metabolic Stability

The in vivo stability was determined in healthy male Swiss albino mice (12 mice in total, body weights 30 ± 5 g). Briefly, each radiolabeled peptide was injected as a bolus (100 μL, 11–22 MBq, 3 nmol of total peptide in saline/EtOH 9/1 *v*/*v*) in the tail vein of mice either untreated (controls) or 20 min after having received per os a slurry of an Entresto^®^ pill (individual 12 mg/200 mL doses of pill per animal; Entresto^®^-group; Novartis, Basel, Switzerland), as previously described [34,35]. Blood was collected 5 min post-injection (pi) in a chilled syringe directly from the heart (0.5–1 mL) and samples were processed prior to being analyzed by HPLC for the detection of forming radiometabolites, as previously described [25,35]. For analyses, an XBridge Shield RP18 (5 μm, 4.6 mm × 20 mm) column (Waters, Vienna, Austria) was eluted at a flow rate of 1 mL/min with 0.1% TFA in H_2_O (A) and MeCN (B) with the following linear gradient system: 100%A/0%B at 0 min, with B linearly increasing by 1%/min to 60%A/40%B (system 2). The *t*_R_ of the intact radiopeptide was determined by coinjection with the respective [^111^In]In-DOTAGA-PEG_2_-RM26 (*t*_R_ = 30 min)/[^111^In]In-AU-RM26-M1 (*t*_R_ = 29 min) reference in the HPLC.

### 2.5. Cell Binding and Internalization

The binding of radiolabeled peptides to GRPR was tested in PC-3 cells (GRPR positive). Peptides were added to cells (10^6^ cells/well) in concentration of 2 nM, with or without pre-blocking of receptors with 1 µM of NOTA-PEG_2_-RM26 for 10 min at room temperature. After incubation for 1 h at 37 °C, the cells were detached, collected, and measured for their activity content.

The internalization assay was performed at 1, 2, 6, and 24 h of incubation with 2 nM solution of radiolabeled peptides as previously described [36]. All in vitro experiments were performed in triplicates.

### 2.6. Affinity Measurements

The dissociation constants, (K_D,_ affinity) for [^111^In]In-DOTAGA-PEG_2_-RM26 and [^111^In]In-AU-RM26-M1, were determined by real-time measurements using the LigandTracer^®^ yellow (Ridgeview Diagnostics, Uppsala, Sweden), as described earlier [31]. PC-3 cells (3 × 10^6^ cells/petri dish) were incubated at room temperature with radiolabeled peptides in concentrations of 1 and 3 nM. The association and dissociation constants (k_on_ and k_off_) were determined using TraceDrawer Software (Ridgeview Instruments, Vänge, Sweden).

### 2.7. In Vivo Experiments

Biodistribution was conducted in Balb/c nu/nu mice (Scanbur A/S, Stockholm, Sweden), implanted subcutaneously in their flanks with PC-3 cells (6 × 10^6^ cells/mouse) 3–4 weeks before the study. Mice in groups of 4 for each data point were injected in the tail vein with either [^111^In]In-DOTAGA-PEG_2_-RM26 or [^111^In]In-AU-RM26-M1 (100 μL, 30 kBq, 40 pmol of total peptide in saline/EtOH 9/1 *v*/*v*); animals were euthanized at 1, 4, and 24 h pi, and biodistribution was conducted. An extra animal group received, in addition to [^111^In]In-AU-RM26-M1, an excess of unlabeled NOTA-PEG_2_-RM26 (5 nmol) to determine non-specific tumor uptake (in vivo GRPR-blocking). An additional negative control mice group (Balb/c nu/nu mice implanted with 6 × 10^6^ DU-145 cells; GRPR negative, n = 4) was included to evaluate the GRPR-targeting specificity of [^111^In]In-AU-RM26-M1. Biodistributions were conducted at 1 h pi for both control groups.

### 2.8. SPECT/CT Imaging

The PC-3 tumor-bearing Balb/c nu/nu mice were injected with 1 MBq (40 pmol) of [^111^In]In-AU-RM26-M1 and imaged at 1, 4, and 24 h pi on the nanoScan^®^ SPECT/CT system from Mediso Medical Imaging Systems (Budapest, Hungary). SPECT raw data were reconstructed using Tera-Tomo™ 3D SPECT reconstruction technology (Mediso Medical Imaging Systems Ltd., Budapest, Hungary). CT data were reconstructed using Filter Back Projection in Nucline 2.03 Software (Mediso Medical Imaging Systems Ltd., Budapest, Hungary). SPECT and CT files were fused using the Nucline 2.03 Software and are presented as maximum intensity projections in the RGB color scale.

## 3. Results

### 3.1. Radiolabeling and Radiochemical Stability

**Figure 2:**
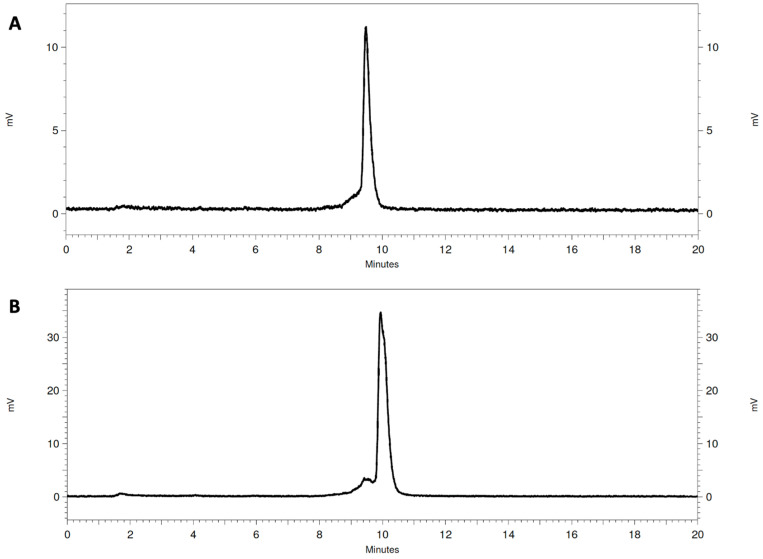
Representative radio-HPLC chromatogram of [^111^In]In-AU-RM26-M1 (**A**) and [^111^In]In-DOTAGA-PEG_2_-RM26 (**B**) (system 1).

The GRPR antagonists were radiolabeled with In-111 in high radiochemical yields (>98%), as determined with ITLC and RP-HPLC (Figure 2). The presence of small additional peaks could be explained by the use of an enantiomeric mixture of R- and S-DOTAGA for the synthesis of the studied peptides [37]. The molar activity of the labeled peptides was 13.3 MBq/nmol. The radiometal complexes were stable during competition with EDTA and when incubated with human sera (Table 1).

### 3.2. Cell Binding and Internalization

**Figure 3:**
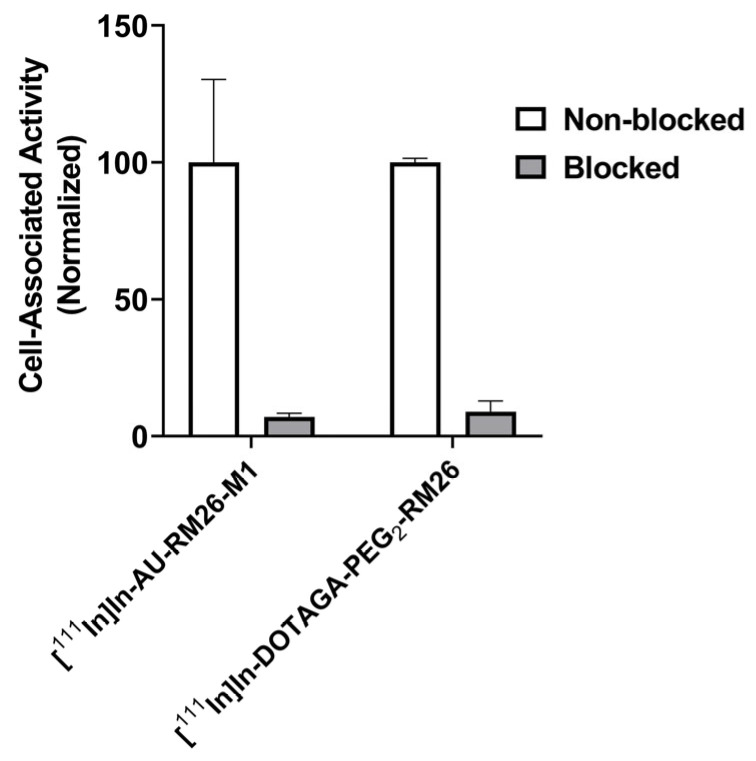
GRPR-specificity of [^111^In]In-AU-RM26-M1 and [^111^In]In-DOTAGA-PEG_2_-RM26 in PC-3 cells. For blocking, a 100-fold molar excess of non-labeled NOTA-RM26 was added before addition of a radioligand. The final concentration of radiolabeled compounds was 2 nM. The data are presented as average ± standard deviation of three samples.

**Figure 4:**
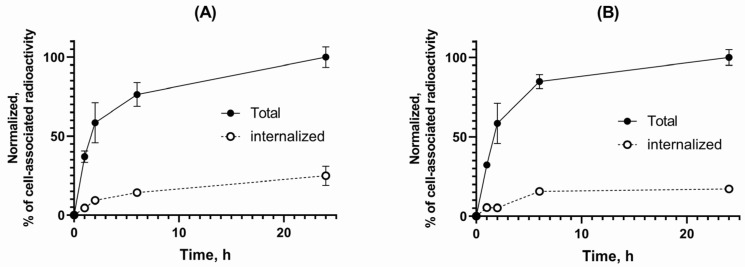
Internalization assay results of [^111^In]In-AU-RM26-M1 (**A**) and [^111^In]In-DOTAGA-PEG_2_-RM26 (**B**) at 1, 2, 6, and 24 h of incubation at 37 °C. The data represent the percentage of cell-associated activity, normalized to the highest uptake. The error bars represent the standard deviation.

Both radiolabeled GRPR antagonists showed a significantly higher uptake in PC-3 cells when incubated alone than in the presence of excess GRPR-blocker (Figure 3). The internalization of both radiolabeled GRPR antagonists was slow as shown in Figure 4. [^111^In]In-AU-RM26-M1 had a higher internalized fraction (25 ± 6%) than [^111^In]In-DOTAGA-PEG_2_-RM26 (17 ± 1%) after 24 h of incubation. Data for the internalization pattern of [^111^In]In-DOTAGA-PEG_2_-RM26 were in good agreement with previous studies [30]. The cell-associated activity was similar for both tested peptides: for [^111^In]In-AU-RM26-M1, cell-associated activity was 6.9 ± 0.7% from added activity at 1 h of incubation and 19 ± 1% at 24 h of incubation; for [^111^In]In-DOTAGA-PEG_2_-RM26—8.3 ± 0.2% and 21.8 ± 0.7%, respectively.

### 3.3. Affinity Measurements

**Figure A1:**
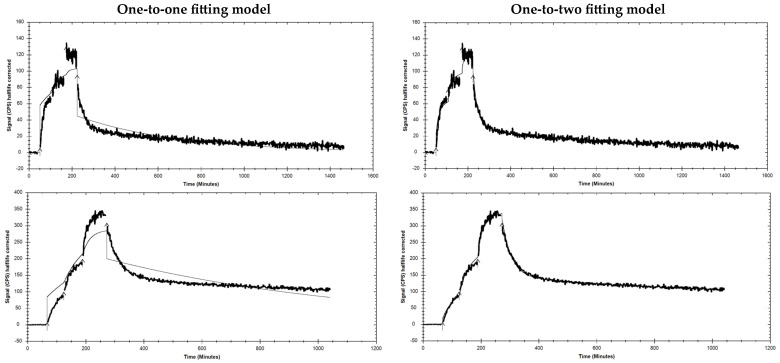
LigandTracer sensorgram of [^111^In]In-AU-RM26-M1 (**upper row**) and [^111^In]In-DOTAGA-PEG_2_-RM26 (**bottom row**) binding to GRPR-expressing PC-3 cells. One-to-one (**left curves**) and one-to-two (**right curves**) fitting models were applied for analyses.

The measured rates of association (k_on_) and dissociation (k_off_) of each radiolabeled peptide to GRPR-positive PC-3 cells were analyzed using one-to-one and one-to-two fitting models, and the last one demonstrated the best fitting for both radiolabeled peptides (Figure A1). The affinity measurements of the radiolabeled GRPR antagonists showed dissociation constant (K_D_) values in the low nanomolar range, as shown in Table 2.

### 3.4. In Vivo Studies

**Figure 5:**
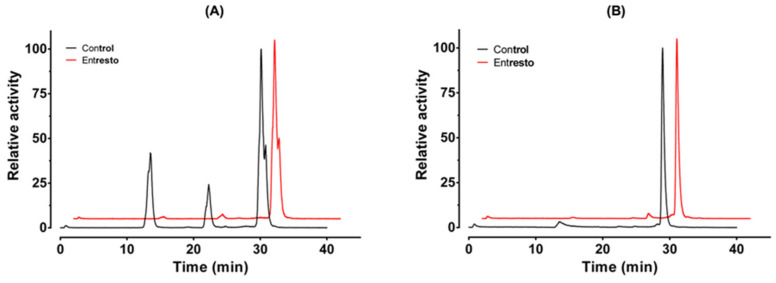
Metabolic stability of [^111^In]In-DOTAGA-PEG_2_-RM26 (**A**) and [^111^In]In-AU-RM26-M1 (**B**) in peripheral mice blood at 5 min pi (system 2). Control group: black line; Entresto^®^-group: red line.

The metabolic stability study revealed a 69 ± 2% of [^111^In]In-DOTAGA-PEG_2_-RM26 remaining intact in peripheral mice blood at 5 min pi for controls (Figure 5A). The respective value in the Entresto^®^-treated group was found to be significantly higher (98.3 ± 0.7% intact; *p* < 0.0001). These data were in good agreement with the published data for [^111^In]In-DOTAGA-PEG_2_-RM26 [31]. In the case of Sar^11^-substituted [^111^In]In-AU-RM26-M1, the percentage of intact radioligands in the control group was significantly higher than the reference, [^111^In]In-DOTAGA-PEG_2_-RM26 (88 ± 8% intact at 5 min pi; *p* < 0.0001, n = 3). This value slightly, but not significantly, increased in the Entresto^®^-treated group (91 ± 2% intact at 5 min pi; *p* > 0.05) (Figure 5B).

**Figure 6:**
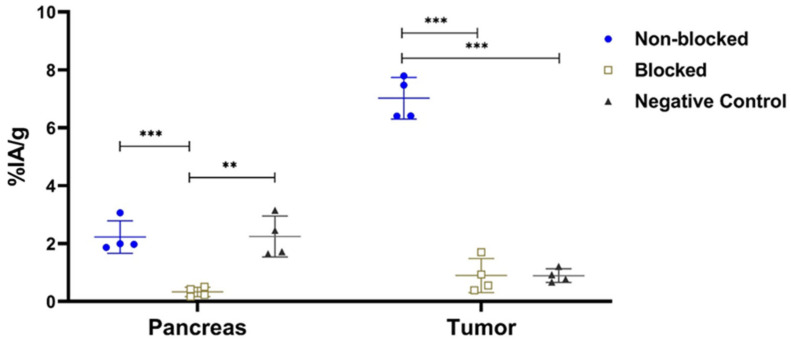
The in vivo GRPR-targeting specificity results for [^111^In]In-AU-RM26-M1. Data were collected at 1 h pi. The positive control (GRPR-positive tumors) data are presented using the blue circles; the GRPR-blocked group is presented using the empty golden squares, and the negative control mice group (GRPR-negative tumors) is presented using the black triangles. Data represent average %IA/g ± sd, n = 4, in either pancreas or tumor; ** denote a *p* < 0.01, *** denote a *p* < 0.001.

The in vivo GRPR-targeting specificity study for [^111^In]In-AU-RM26-M1 in mice bearing PC-3 tumors (GRPR-positive) showed a significant reduction in tumor activity uptake in the GRPR-blocking group at 1 h pi (0.9 ± 0.6%IA/g vs. 7.0 ± 0.7%IA/g for controls; *p* < 0.001, n = 4). The radioligand uptake in the DU-145 tumors (GRPR-negative) was also significantly lower than in the positive control group at the same time point (0.9 ± 0.2%IA/g vs. 7.0 ± 0.7%IA/g; *p* < 0.001, n = 4). Furthermore, the uptake in the pancreas (GRPR-positive organ) for the positive control group was 2.2 ± 0.6%IA/g, for the negative control group was 2.2 ± 0.7%IA/g, and for the blocked group was 0.3 ± 0.2%IA/g, with statistically significant differences between each control group and the blocked group, as presented in Figure 6.

**Figure 7:**
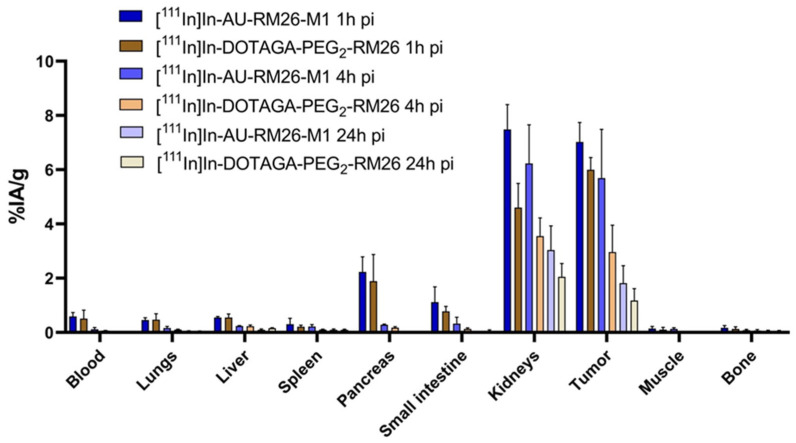
Biodistribution results of [^111^In]In-AU-RM26-M1 (blue bars) and [^111^In]In-DOTAGA-PEG_2_-RM26 (brown bars) at 1, 4, and 24 h pi. The data represent average %IA/g ± SD, n = 4.

**Figure 8:**
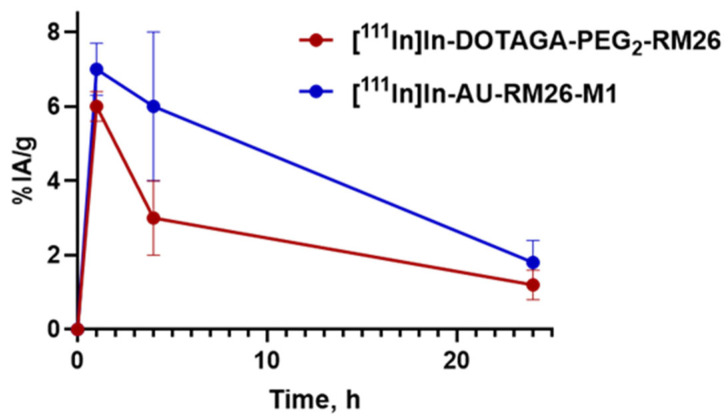
Overall uptake of [^111^In]In-AU-RM26-M1 (blue line) and [^111^In]In-DOTAGA-PEG2-RM26 (red line) in PC-3 tumors in Balb/c nu/nu mice over time. The values represent average uptake ± SD, n = 4.

Comparative biodistribution results for [^111^In]In-AU-RM26-M1 and [^111^In]In-DOTAGA-PEG_2_-RM26 (Figure 7 and Table A1) in tumor-bearing mice at 1, 4, and 24 h pi reveal a similar profile. While the majority of examined healthy organs had low activity uptake already at 1 h pi, tumors, kidneys, pancreas and—to a lesser extent—intestinal wall, displayed the highest background levels. Tumor and kidney uptake was higher for the Sar^11^-modified analog [^111^In]In-AU-RM26-M1 compared with the Gly^11^-reference [^111^In]In-DOTAGA-PEG_2_-RM26. Thus, the uptake was significantly higher at 1 and 4 h pi for kidneys and at 4 h pi for tumors. The area under the tumor uptake curve (AUC, Figure 8) was about twice as high for the metabolically stabilized [^111^In]In-AU-RM26-M1 as for the reference (98 ± 21 vs. 56 ± 11, *p* < 0.0125).

The tumor-to-organ ratios for [^111^In]In-DOTAGA-PEG_2_-RM26 and [^111^In]In-AU-RM26-M1 at the three investigated time points were comparable with minimal differences, as summarized in Table A2.

**Figure 9:**
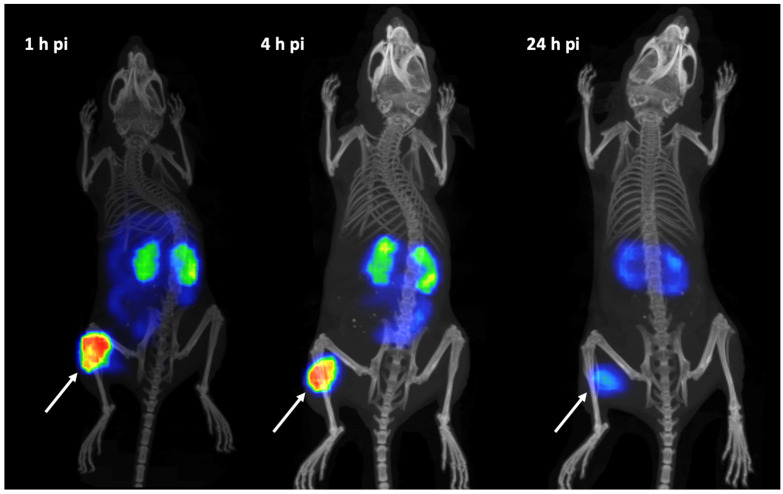
SPECT/CT images of PC-3 tumor-bearing Balb/c nu/nu mice at 1, 4, and 24 h pi of [^111^In]In-AU-RM26-M1. The white arrows point to the PC-3 xenografts.

SPECT/CT imaging of [^111^In]In-AU-RM26-M1 in PC-3 tumor-bearing mice showed clear visualization of tumor uptake at 1, 4, and 24 h pi (Figure 9). No other organs displayed any detectable uptake except for the kidneys. The SPECT images were in agreement with ex vivo biodistribution data.

## 4. Discussion

The high incidence and mortality of prostate cancer necessitate the development of new, improved theranostic options. Due to the limitations of PSMA-targeting, other prostate cancer cell markers, including GRPR, need to be further explored. Numerous GRPR radioantagonists have been developed and evaluated thus far, showing great potential in prostate cancer management [28]. The metabolic stability of circulating peptide radioligands was shown to be an essential factor for GRPR-targeting of pathological lesions, improving imaging quality and radiotherapy output [25,27,38]. High in vivo stability of radiopeptide has resulted in a higher tumor uptake and, in most cases, improved tumor-to-background ratios. The major protease involved in the rapid in vivo degradation of bombesin-like peptides (both GRPR agonists and antagonists) in blood circulation is the ectoenzyme NEP, the action of which remained long elusive [24,25,26,39].

Like other peptides, peptide-based GRPR antagonists are prone to the action of proteases that cleave specific peptide bonds in the chain. For peptide radioligands in circulation, this action translates into a lower percentage of intact peptides reaching the target following intravenous administration [24]. Many attempts have been made over the years to improve the metabolic stability of GRPR antagonists with the substitution of selected amino acids, such as Gly^11^ and Gln^7^ by unnatural residues [28]. In this study, we introduce AU-RM26-M1 generated with the Gly^11^/Sar^11^-substitution in DOTAGA-PEG_2_-RM26 (Figure 1), previously reported for good therapeutic efficacy in a murine model [31]. The same substitution previously led to the SPECT radiotracer [^99m^Tc]Tc-DB15 showing an attractive profile in GRPR-positive lesions in animal models and in breast cancer patients [32,33]. It should be noted that other Gly^11^-replacements have not been equally successful. For example, [^111^In]In-SB4, where Gly^11^ is replaced by DAla^11^, despite its high in vivo metabolic stability demonstrated lower GRPR-affinity and cell uptake, eventually translating into an inferior tumor uptake compared with the unmodified parent [^111^In]In-SB3 [38].

The low and slowly increasing internalized fraction of [^111^In]In-AU-RM26-M1 in PC-3 cells is concordant with a GRPR antagonist profile (Figure 4). This finding indicates that the antagonistic features of [^111^In]In-AU-RM26-M1 were not affected by the adopted Gly^11^/Sar^11^-substitution, as after binding to GRPR the bulk of cell-associated activity remained attached to the cell membrane. However, it is interesting to note that [^111^In]In-AU-RM26-M1 internalized faster compared to the unmodified [^111^In]In-DOTAGA-PEG_2_-RM26, most probably via a non-ligand-induced GRPR cellular recycling process. This feature of [^111^In]In-AU-RM26-M1 may turn out to be advantageous for the therapeutic application of AU-RM26-M1 labeled with beta emitters, such as Lu-177, by prolonging tumor residence times. Indeed, while the initial activity uptake of [^111^In]In-AU-RM26-M1 in the GRPR-positive PC-3 tumors was only slightly higher than the parent radioligand, tumor retention was clearly superior at 4 h pi, as shown by the two-fold higher tumor values (Figure 7 and Table A1).

The real-time receptor affinity measurements revealed a comparably high affinity for both radioligands to GRPR, with equilibrium dissociation constants found in the low nanomolar range (Table 2). Interestingly, the interaction of both peptides with GRPR measured in real time on living PC-3 cells fits better to the one-to-two model than to the one-to-one model (Figure A1). A single value of 0.44 ± 0.05 nM (one-to-one fitting) was previously reported for [^111^In]In-DOTAGA-PEG_2_-RM26 [30]. However, when the GRPR affinity of the closely related antagonist [^111^In]In-NOTA-PEG_6_-RM26 was studied in more detail in similar experimental settings, two different interactions were found [40]. The authors hypothesized that either the GRPR is present in two conformations on PC-3 cells, or a conformation change occurs after the formation of the receptor–ligand complex. In the second case, k_a1_ and k_a2_ should have close values. We can speculate that in this study, we observed the presence of two conformations rather than a conformation change (Table 2). The coexistence of two types of binding interactions could decrease the radioligand uptake in target cells if these interaction types are considerably different in strength. However, the two dissociation constants measured for [^111^In]In-AU-RM26-M1 were not markedly different (0.53 ± 0.46 and 1.6 ± 0.3 nM).

Our initial hypothesis that the Gly^11^/Sar^11^-substitution may enhance the resistance of BBN-like radioligands to the rapid proteolytic action of NEP was confirmed using the comparative in vivo stability assay performed for [^111^In]In-DOTAGA-PEG_2_-RM26 and its Sar^11^-version, [^111^In]In-AU-RM26-M1 (Figure 5). Thus, about 30% of [^111^In]In-DOTAGA-PEG_2_-RM26 was degraded in peripheral mice blood already at 5 min pi, while in situ inhibition of NEP by pre-administration of Entresto^®^ per os led to stabilization of the radioligand in this time interval. It should be noted that the commercially available drug Entresto^®^ contains the prodrug sacubitril, in vivo releasing the selective and potent NEP inhibitor sacubitrilat [34,35]. This finding pin-points NEP as the major degrading protease of [^111^In]In-DOTAGA-PEG_2_-RM26, in agreement with previous findings with other BBN-like radioligands [24,25,27,38]. Remarkably, no significant difference was found in the stability of [^111^In]In-AU-RM26-M1 between untreated mice and those treated with sacubitril (Entresto^®^), suggesting that Gly^11^/Sar^11^-subsitution rendered [^111^In]In-AU-RM26-M1 resistant to NEP, in line with previous reports [33]. Overall, the in vivo stability of [^111^In]In-AU-RM26-M1 was significantly better than the parent [^111^In]In-DOTAGA-PEG_2_-RM26. This is an advantageous quality of [^111^In]In-AU-RM26-M1 when clinical translation is considered because it facilitates its clinical use without the need for co-medication.

The new Sar^11^-modified [^111^In]In-AU-RM26-M1 successfully targeted the experimental PC-3 tumors and the GRPR-rich pancreas in mice via a GRPR-specific process, as verified by the significant decrease in tumor and pancreas uptake in the mice that received a high excess of a GRPR-specific peptide for an in vivo GRPR blockade. The specificity of the PC-3 tumor uptake (GRPR-positive) was further confirmed by the significantly lower uptake of the radioligand in the DU-145 tumors (GRPR-negative) that was at the same level as the GRPR-blocked group (Figure 6). In contrast, the pancreatic uptake was indistinguishable between the groups of PC-3 and DU-145 tumor-bearing mice. As expected, in both groups pancreatic uptake was found significantly higher than in the GRPR-blocked group of PC-3 tumor-bearing mice. The SPECT/CT images of tumor-bearing mice after injection of [^111^In]In-AU-RM26-M1 were in agreement with the biodistribution profile, with clear visualization of the GRPR-positive tumors (Figure 9).

The comparison between the biodistribution of [^111^In]In-AU-RM26-M1 and [^111^In]In-DOTAGA-PEG2-RM26 over time indicated a tendency of the Sar^11^-radioligand for improved GRPR-mediated tumor uptake. Accordingly, a slower wash out of radiolabeled peptide (Figure 8) is expected to translate to an increased absorbed dose for tumors and be beneficial for radiotherapy.

Overall, tumor-to-organ ratios were higher for [^111^In]In-AU-RM26-M1 than for [^111^In]In-DOTAGA-PEG_2_-RM26 due to better activity retention in tumors (Table A2). Interestingly, however, no statistically significant differences were found in the tumor uptake between the two radioligands at 1 and 24 h pi. On the other hand, [^111^In]In-AU-RM26-M1 displayed a higher kidney uptake than [^111^In]In-DOTAGA-PEG_2_-RM26 with minor uptake differences in the rest of the organs. Overall, the biodistribution profile of [^111^In]In-AU-RM26-M1 has been improved as far as tumor uptake and tumor-to-organ ratios are concerned, with the exception of kidney uptake. Indeed, the biodistribution profile of [^111^In]In-AU-RM26-M1 (as a prototype to the agent for targeted radiotherapy) has not shown marked improvements, taking into account the tumor-to-kidney ratios in our mice model, despite the enhanced metabolic stability achieved. Certainly, extrapolation of biodistribution results from In-111 to Lu-177, as well as from mice to humans should be made with caution. In general, renal excretion of radiolabeled peptides has been shown to involve reabsorption in the proximal tubules, as a part of a mechanism aimed to prevent the excessive loss of proteins. Several systems and processes responsible for reabsorption have been identified thus far, such as the megalin/cubilin endocytic receptor system, pinocytosis, organic anion, organic cation, and oligopeptide transporter families [41]. Coinfusion of positively charged amino acids (lysine/arginine) and negatively charged succinylated gelatin (Gelofusine) are routinely applied to decrease the reabsorption of radiopeptides used clinically for targeted radiotherapy [41]. The optimization of injected peptide amount is another way to increase the ratio of absorbed dose in tumors to that in kidneys. For example, kidney uptake decreased two-fold when the peptide dose for GRPR-targeting peptide [^177^Lu]Lu-NeoB increased from 400 to 1200 pmol [42].

## 5. Conclusions

In this study, we reported the effects of Gly^11^/Sar^11^-substitution on the performance of the GRPR-antagonist-based radioligand [^111^In]In-AU-RM26-M1 after a head-to-head comparison with the [^111^In]In-DOTAGA-PEG_2_-RM26 parent. This modification resulted in significant improvement in metabolic stability of circulating [^111^In]In-AU-RM26-M1 and translated into better uptake and retention in a GRPR-positive tumor mice model. However, the overall biodistribution profile of [^111^In]In-AU-RM26-M1 was not notably better, especially as far as kidney uptake in mice is concerned. These results call for further modifications with the aim of decreasing the renal uptake and retention while maintaining the high metabolic stability achieved for [^111^In]In-AU-RM26-M1, especially when radionuclide therapy with beta or alpha emitters is considered.

## Figures and Tables

**Table 1:** Radiochemical stability for [^111^In]In-AU-RM26-M1 and [^111^In]In-DOTAGA-PEG_2_-RM26 presented as % protein-associated activity (average ± SD, n = 3); incubation: 1 h at 37 °C.

Radiopeptide	PBS	EDTA	Serum
[^111^In]In-AU-RM26-M1	100.0 ± 0.3	98.7 ± 0.3	99.2 ± 0.3
[^111^In]In-DOTAGA-PEG_2_-RM26	99.90 ± 0.06	96 ± 3	99.9 ± 0.2

**Table 2:** The results of the real-time affinity measurements of PC-3 cells. The values for k_a_ and k_d_ were determined from the one-to-two fitting model.

Radiopeptide	k_a1_ (M^−1^ s^−1^)	k_d1_ (s^−1^)	K_D1_ (nM)	k_a2_ (M^−1^ s^−1^)	k_d2_ (s^−1^)	K_D2_ (nM)
[^111^In]In-AU-RM26-M1	5.7 ± 5.6 × 10^4^	1.7 ± 3.3 × 10^−6^	0.53 ± 0.46	3.9 ± 2.3 × 10^5^	5.7 ± 2.6 × 10^−4^	1.6 ± 0.3
[^111^In]In-DOTAGA-PEG_2_-RM26	8.54 × 10^4^	9.73 × 10^−6^	0.11	3.03 × 10^5^	5.97 × 10^−4^	1.97

## Data Availability

The data generated during the current study are available from the corresponding author upon reasonable request.

## Appendix A

**Table A1:** Biodistribution results of [^111^In]In-AU-RM26-M1 and [^111^In]In-DOTAGA-PEG_2_-RM26 at 1, 4, and 24 h pi in Balb/c nu/nu mice bearing PC-3 tumors, expressed as average %IA/g ± sd, n = 4.

	%IA/g					
Variant Organ	[^111^In]In-AU-RM26-M1			[^111^In]In-DOTAGA-PEG_2_-RM26		
	1 h	4 h	24 h	1 h	4 h	24 h
Blood	0.6 ± 0.1	0.11 ± 0.07	0.02 ± 0.01	0.5 ± 0.3	0.07 ± 0.01	0.015 ± 0.003
Lungs	0.46 ± 0.09	0.17 ± 0.06	0.03 ± 0.03	0.5 ± 0.2	0.11 ± 0.02	0.04 ± 0.01
Liver	0.55 ± 0.04	0.24 ± 0.02	0.10 ± 0.03 ^c^	0.5 ± 0.1	0.23 ± 0.05	0.16 ± 0.02
Spleen	0.3 ± 0.2	0.22 ± 0.07 ^b^	0.10 ± 0.03	0.21 ± 0.06	0.10 ± 0.02	0.10 ± 0.03
Pancreas	2.23 ± 0.56	0.29 ± 0.02 ^b^	0.03 ± 0.01	1.9 ± 1.0	0.18 ± 0.04	0.03 ± 0.01
Small intestine	1.1 ± 0.6	0.3 ± 0.2	0.03 ± 0.01	0.8 ± 0.2	0.13 ± 0.04	0.05 ± 0.05
Kidneys	7.2 ± 0.9 ^a^	6 ± 1 ^b^	3.0 ± 0.9	4.6 ± 0.9	3.5 ± 0.7	2.0 ± 0.5
Tumor	7.0 ± 0.7	6 ± 2 ^b^	1.8 ± 0.6	6.0 ± 0.4	3.0 ± 1.00	1.2 ± 0.4
Muscle	0.15 ± 0.08	0.13 ± 0.05 ^b^	0.01 ± 0.01	0.11 ± 0.08	0.04 ± 0.01	0.01 ± 0.01
Bone	0.17 ± 0.09	0.09 ± 0.03	0.06 ± 0.03	0.13 ± 0.08	0.07 ± 0.04	0.04 ± 0.03

^a^ indicates a significant difference between [^111^In]In-AU-RM26-M1 and [^111^In]In-DOTAGA-PEG_2_-RM26 at 1 h pi, ^b^ at 4 h pi, and ^c^ at 24 h pi.

**Table A2:** The tumor-to-organ ratios for [^111^In]In-AU-RM26-M1 and [^111^In]In-DOTAGA-PEG_2_-RM26 at 1, 4, and 24 h pi in Balb/c nu/nu mice bearing PC-3 tumors. The values represent average tumor-to-organ ratios ± SD, n = 4.

	Tumor-to-Organ Ratios					
Variant Organ	[^111^In]In-AU-RM26- M1			[^111^In]In-DOTAGA-PEG_2_-RM26		
	1 h	4 h	24 h	1 h	4 h	24 h
Blood	12 ± 3	63 ± 38	119 ± 54	15 ± 6	43 ± 16	79 ± 34
Lungs	16 ± 31	38 ± 19	77 ± 62	15 ± 5	29 ± 11	30 ± 18
Liver	13 ± 2	24 ± 6	21 ± 16	11 ± 3	14 ± 6	7 ± 2
Spleen	31 ± 16	30 ± 17	21 ± 11	30 ± 8	30 ± 12	12± 3
Pancreas	3.3 ± 0.8	20 ± 6	64 ± 20	4 ± 2	17 ± 8	46 ± 15
Small intestine	7 ± 3	22 ± 11	76 ± 17 ^b^	8 ± 2	26 ± 14	28± 14
Kidneys	0.9 ± 0.1 ^a^	0.9 ± 0.3	0.6 ± 0.3	1.3 ± 0.3	0.9 ± 0.4	0.6 ± 0.2
Muscle	57 ± 24	47 ± 16	206 ± 97	78 ± 36	74 ± 20	91 ± 72
Bone	51 ± 24	74 ± 34	34 ± 12	61 ± 33	72 ± 65	21 ± 3

^a^ indicates a significant difference between [^111^In]In-AU-RM26-M1 and [^111^In]In-DOTAGA-PEG_2_-RM26 at 1 h pi and ^b^ at 24 h pi.
